# 6,7-Dihydro-3*H*-1,4-diazepino[1,2,3,4-*lmn*][1,10]phenanthroline-3,9(5*H*)-dione

**DOI:** 10.1107/S1600536810024761

**Published:** 2010-06-30

**Authors:** Said Nadeem, Itrat Anis, Donald VanDerveer, Muhammad Raza Shah

**Affiliations:** a H.E.J. Research Institute of Chemistry, International Center for Chemical and Biological Sciences, University of Karachi, Karachi 75270, Pakistan; b Department of Chemistry, University of Karachi, Karachi 75270, Pakistan; c Chemistry Department, Clemson University, Clemson, SC 29634-0973, USA

## Abstract

In the title compound, C_15_H_12_N_2_O_2_, the seven-membered ring bearing the three methyl­ene C atoms displays a puckered conformation, with the methyl­ene C atoms deviating from the plane of the benzene ring by 0.05 (1), 0.98 (1) and 1.04 (1) Å. The phenanthroline unit is not planar; the dihedral angles between this benzene ring and the other pyridyl rings are 9.62 (4) and 9.31 (4)°. The crystal packing is stabilized by π–π inter­actions between two phenanthroline ring systems, forming a centrosymmetric dimer with a centroid–centroid distance of 3.656 (1) Å.

## Related literature

For background to π–π inter­actions in supra­molecular chemistry, see: Sisson *et al.* (2006 ▶). For a related structure, see: Nadeem *et al.* (2009 ▶).
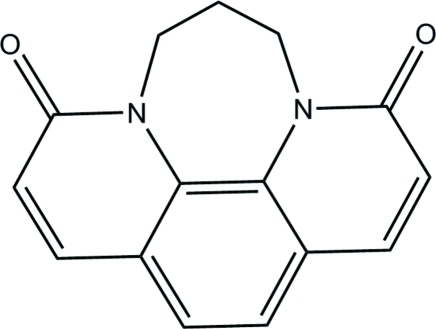


## Experimental

### 

#### Crystal data

C_15_H_12_N_2_O_2_
*M* *_r_* = 252.27
Monoclinic, 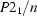

*a* = 9.1853 (18) Å
*b* = 13.931 (3) Å
*c* = 9.4956 (19) Å
β = 111.14 (3)°
*V* = 1133.3 (4) Å^3^
*Z* = 4
Mo *K*α radiation
μ = 0.10 mm^−1^
*T* = 158 K
0.50 × 0.46 × 0.38 mm

#### Data collection

Rigaku Mercury CCD diffractometer
Absorption correction: multi-scan (*REQAB*; Jacobson, 1998 ▶) *T*_min_ = 0.952, *T*_max_ = 0.963
8367 measured reflections
2309 independent reflections
2115 reflections with *I* > 2σ(*I*)
*R*_int_ = 0.016

#### Refinement

*R*[*F* ^2^ > 2σ(*F* ^2^)] = 0.041
*wR*(*F* ^2^) = 0.111
*S* = 1.06
2309 reflections
172 parameters
H-atom parameters constrained
Δρ_max_ = 0.23 e Å^−3^
Δρ_min_ = −0.23 e Å^−3^

### 

Data collection: *CrystalClear* (Rigaku/MSC, 2006 ▶); cell refinement: *CrystalClear*; data reduction: *CrystalClear*; program(s) used to solve structure: *SHELXS97* (Sheldrick, 2008 ▶); program(s) used to refine structure: *SHELXL97* (Sheldrick, 2008 ▶); molecular graphics: *SHELXTL* (Sheldrick, 2008 ▶); software used to prepare material for publication: *SHELXTL* and *PLATON* (Spek, 2009 ▶).

## Supplementary Material

Crystal structure: contains datablocks I, global. DOI: 10.1107/S1600536810024761/ng2793sup1.cif

Structure factors: contains datablocks I. DOI: 10.1107/S1600536810024761/ng2793Isup2.hkl

Additional supplementary materials:  crystallographic information; 3D view; checkCIF report

## Figures and Tables

**Fig. 1.**
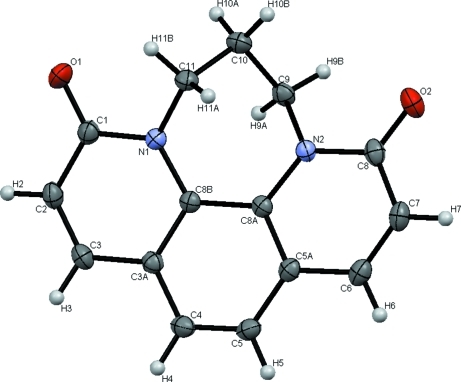
Molecular Structure of (I) with atom labels and 50% probability displacement ellipsoids.

## supplementary crystallographic information

### Comment

In supramolecular chemistry it is well establish that the self-association of individual molecules can lead to the formation of highly complex and fascinating supramolecular assemblies if π–π interactions contribute to the formation of specific motifs (Sisson *et al.*, 2006). As a part of our ongoing investigation on the nature of π-π (Nadeem *et al.*, 2009) stacking and supramolecular chemistry, the title compound (Figure-1), has been prepared and its crystal structure is reported here. The crystal packing is stabilized by π-π interactions between two phenanthroline ring systems forming a centrosymmetric dimer with a centroid···centroid distance of 3.656 (1) Å. A typical double bond distance 1.236 (2) Å was observed for C1—O1 C8—O2 while a characteristic single bond distances 1.386 (1) Å and 1.393 (1) Å were observed for C1—N1 and C8—N2 respectively.

### Experimental

To an ice-cooled solution of potassium hexacyanoferrate(III) (58.6g) and sodium hydroxide (26.8g) in water (100ml) were added in small portions a solution of 6,7-dihydro-5H-[1,4]diazepino[1,2,3,4-lmn][1,10]phenanthroline-4,8-diium bromide (7.6g) in water (50ml),maintaining the temperature under 278 K for 10 minutes. The resulting mixture was neutralized by a dropwise addition of concentrated hydrochloric acid at 273 K. The residual brown pasty material was extracted three times with chloroform, subjected to column chromatography (DCM:MeOH; 100ml:1ml v/v), evaporated to dryness, recrystallization from methanol afforded 1.5g (30%) of title compound as pale yellow needles.

### Refinement

The H atoms were geometrically placed and treated as riding atoms with C—H = 0.96 Å, and *U*_iso_(H) = 1.2 *U*_eq_ (parent C-atom).

### Crystal data

**Table:**

C_15_H_12_N_2_O_2_	*F*(000) = 528
*M**_r_* = 252.27	*D*_x_ = 1.478 Mg m^−^^3^
Monoclinic, *P*2_1_/*n*	Mo *K*α radiation, λ = 0.71073 Å
*a* = 9.1853 (18) Å	Cell parameters from 3526 reflections
*b* = 13.931 (3) Å	θ = 2.7–26.4°
*c* = 9.4956 (19) Å	µ = 0.10 mm^−^^1^
β = 111.14 (3)°	*T* = 158 K
*V* = 1133.3 (4) Å^3^	Chip, yellow
*Z* = 4	0.50 × 0.46 × 0.38 mm

### Data collection

**Table:**

Rigaku Mercury CCD diffractometer	2309 independent reflections
Radiation source: Sealed Tube	2115 reflections with *I* > 2σ(*I*)
Graphite Monochromator	*R*_int_ = 0.016
Detector resolution: 14.6306 pixels mm^-1^	θ_max_ = 26.4°, θ_min_ = 2.7°
ω scans	*h* = −11→9
Absorption correction: multi-scan (*REQAB*; Jacobson, 1998)	*k* = −17→17
*T*_min_ = 0.952, *T*_max_ = 0.963	*l* = −10→11
8367 measured reflections	

### Refinement

**Table:**

Refinement on *F*^2^	Primary atom site location: structure-invariant direct methods
Least-squares matrix: full	Secondary atom site location: difference Fourier map
*R*[*F*^2^ > 2σ(*F*^2^)] = 0.041	Hydrogen site location: inferred from neighbouring sites
*wR*(*F*^2^) = 0.111	H-atom parameters constrained
*S* = 1.06	*w* = 1/[σ^2^(*F*_o_^2^) + (0.0632*P*)^2^ + 0.3698*P*] where *P* = (*F*_o_^2^ + 2*F*_c_^2^)/3
2309 reflections	(Δ/σ)_max_ < 0.001
172 parameters	Δρ_max_ = 0.23 e Å^−^^3^
0 restraints	Δρ_min_ = −0.23 e Å^−^^3^

### Special details

**Table:**

Geometry. All esds (except the esd in the dihedral angle between two l.s. planes) are estimated using the full covariance matrix. The cell esds are taken into account individually in the estimation of esds in distances, angles and torsion angles; correlations between esds in cell parameters are only used when they are defined by crystal symmetry. An approximate (isotropic) treatment of cell esds is used for estimating esds involving l.s. planes.
Refinement. Refinement of F^2^ against ALL reflections. The weighted R-factor wR and goodness of fit S are based on F^2^, conventional R-factors R are based on F, with F set to zero for negative F^2^. The threshold expression of F^2^ > 2sigma(F^2^) is used only for calculating R-factors(gt) etc. and is not relevant to the choice of reflections for refinement. R-factors based on F^2^ are statistically about twice as large as those based on F, and R- factors based on ALL data will be even larger.

### Fractional atomic coordinates and isotropic or equivalent isotropic displacement parameters (Å^2^)

**Table:**

	*x*	*y*	*z*	*U*_iso_*/*U*_eq_	
N1	1.02892 (11)	0.14451 (7)	0.36246 (11)	0.0183 (2)	
N2	0.79115 (11)	0.11065 (7)	0.06165 (11)	0.0186 (2)	
O1	1.09796 (11)	0.14573 (7)	0.61843 (10)	0.0313 (2)	
O2	0.53670 (10)	0.10333 (7)	−0.09893 (10)	0.0285 (2)	
C1	1.13585 (14)	0.13175 (9)	0.50775 (13)	0.0219 (3)	
C2	1.29358 (14)	0.10579 (9)	0.52135 (13)	0.0239 (3)	
H2	1.3694	0.0911	0.6190	0.029*	
C3	1.33514 (14)	0.10204 (9)	0.39944 (14)	0.0234 (3)	
H3	1.4423	0.0905	0.4128	0.028*	
C3A	1.22264 (13)	0.11496 (8)	0.24970 (13)	0.0193 (3)	
C4	1.26651 (14)	0.11749 (8)	0.12166 (14)	0.0228 (3)	
H4	1.3735	0.1075	0.1328	0.027*	
C5	1.15645 (14)	0.13419 (9)	−0.01825 (13)	0.0223 (3)	
H5	1.1879	0.1443	−0.1032	0.027*	
C5A	0.99677 (14)	0.13661 (8)	−0.03786 (13)	0.0197 (3)	
C6	0.87822 (15)	0.14730 (9)	−0.18533 (13)	0.0238 (3)	
H6	0.9079	0.1576	−0.2713	0.029*	
C7	0.72644 (15)	0.14297 (9)	−0.20391 (13)	0.0250 (3)	
H7	0.6497	0.1561	−0.3018	0.030*	
C8	0.67481 (14)	0.11909 (8)	−0.08064 (14)	0.0216 (3)	
C8A	0.94915 (13)	0.12578 (8)	0.08617 (13)	0.0171 (3)	
C8B	1.06584 (13)	0.12776 (8)	0.23397 (13)	0.0170 (2)	
C9	0.74874 (13)	0.06207 (8)	0.17982 (13)	0.0216 (3)	
H9A	0.8241	0.0129	0.2259	0.026*	
H9B	0.6488	0.0318	0.1341	0.026*	
C10	0.74195 (14)	0.13128 (9)	0.30056 (14)	0.0239 (3)	
H10A	0.7405	0.0961	0.3869	0.029*	
H10B	0.6483	0.1689	0.2625	0.029*	
C11	0.88345 (13)	0.19676 (8)	0.34603 (13)	0.0209 (3)	
H11A	0.8667	0.2460	0.2712	0.025*	
H11B	0.8950	0.2273	0.4401	0.025*	

### Atomic displacement parameters (Å^2^)

**Table:**

	*U*^11^	*U*^22^	*U*^33^	*U*^12^	*U*^13^	*U*^23^
N1	0.0176 (5)	0.0208 (5)	0.0166 (5)	0.0005 (4)	0.0063 (4)	0.0000 (4)
N2	0.0164 (5)	0.0197 (5)	0.0181 (5)	−0.0012 (4)	0.0044 (4)	0.0005 (4)
O1	0.0303 (5)	0.0472 (6)	0.0171 (4)	0.0023 (4)	0.0094 (4)	0.0011 (4)
O2	0.0179 (4)	0.0298 (5)	0.0316 (5)	−0.0017 (3)	0.0015 (4)	0.0023 (4)
C1	0.0220 (6)	0.0245 (6)	0.0178 (5)	−0.0021 (4)	0.0055 (4)	0.0008 (4)
C2	0.0206 (6)	0.0270 (6)	0.0194 (5)	−0.0007 (5)	0.0016 (4)	0.0024 (4)
C3	0.0165 (5)	0.0260 (6)	0.0250 (6)	0.0013 (4)	0.0040 (5)	0.0009 (5)
C3A	0.0183 (6)	0.0187 (5)	0.0204 (6)	−0.0006 (4)	0.0065 (4)	−0.0011 (4)
C4	0.0201 (6)	0.0238 (6)	0.0267 (6)	−0.0010 (4)	0.0112 (5)	−0.0032 (5)
C5	0.0255 (6)	0.0229 (6)	0.0220 (6)	−0.0029 (5)	0.0130 (5)	−0.0027 (4)
C5A	0.0228 (6)	0.0174 (5)	0.0189 (5)	−0.0016 (4)	0.0077 (5)	−0.0009 (4)
C6	0.0296 (6)	0.0231 (6)	0.0177 (6)	−0.0016 (5)	0.0073 (5)	−0.0001 (4)
C7	0.0264 (6)	0.0248 (6)	0.0180 (5)	0.0000 (5)	0.0011 (5)	0.0005 (4)
C8	0.0202 (6)	0.0172 (5)	0.0231 (6)	−0.0003 (4)	0.0026 (5)	−0.0009 (4)
C8A	0.0174 (5)	0.0151 (5)	0.0189 (6)	−0.0007 (4)	0.0065 (4)	−0.0005 (4)
C8B	0.0177 (6)	0.0157 (5)	0.0176 (5)	−0.0007 (4)	0.0063 (4)	0.0001 (4)
C9	0.0188 (5)	0.0225 (6)	0.0236 (6)	−0.0034 (4)	0.0077 (4)	0.0019 (4)
C10	0.0195 (6)	0.0290 (6)	0.0254 (6)	−0.0010 (5)	0.0107 (5)	0.0003 (5)
C11	0.0190 (5)	0.0226 (6)	0.0216 (5)	0.0019 (4)	0.0081 (4)	−0.0011 (4)

### Geometric parameters (Å, °)

**Table:**

N1—C1	1.3866 (16)	C5—C5A	1.4107 (17)
N1—C8B	1.3987 (15)	C5—H5	0.9600
N1—C11	1.4795 (14)	C5A—C8A	1.4044 (16)
N2—C8	1.3929 (16)	C5A—C6	1.4385 (17)
N2—C8A	1.4005 (15)	C6—C7	1.3420 (18)
N2—C9	1.4779 (14)	C6—H6	0.9600
O1—C1	1.2358 (15)	C7—C8	1.4512 (18)
O2—C8	1.2364 (15)	C7—H7	0.9600
C1—C2	1.4531 (17)	C8A—C8B	1.4264 (17)
C2—C3	1.3443 (17)	C9—C10	1.5165 (17)
C2—H2	0.9600	C9—H9A	0.9600
C3—C3A	1.4361 (17)	C9—H9B	0.9600
C3—H3	0.9600	C10—C11	1.5176 (16)
C3A—C8B	1.4048 (16)	C10—H10A	0.9600
C3A—C4	1.4124 (17)	C10—H10B	0.9600
C4—C5	1.3684 (18)	C11—H11A	0.9600
C4—H4	0.9600	C11—H11B	0.9600
			
C1—N1—C8B	122.61 (10)	C6—C7—C8	122.03 (11)
C1—N1—C11	117.18 (10)	C6—C7—H7	119.0
C8B—N1—C11	118.94 (9)	C8—C7—H7	119.0
C8—N2—C8A	122.35 (10)	O2—C8—N2	120.71 (11)
C8—N2—C9	117.07 (9)	O2—C8—C7	123.00 (11)
C8A—N2—C9	118.93 (9)	N2—C8—C7	116.25 (11)
O1—C1—N1	120.68 (11)	N2—C8A—C5A	119.61 (10)
O1—C1—C2	122.76 (11)	N2—C8A—C8B	122.17 (10)
N1—C1—C2	116.50 (10)	C5A—C8A—C8B	118.19 (11)
C3—C2—C1	121.12 (11)	N1—C8B—C3A	119.36 (10)
C3—C2—H2	119.4	N1—C8B—C8A	121.94 (10)
C1—C2—H2	119.4	C3A—C8B—C8A	118.68 (11)
C2—C3—C3A	121.42 (11)	N2—C9—C10	112.13 (10)
C2—C3—H3	119.3	N2—C9—H9A	109.2
C3A—C3—H3	119.3	C10—C9—H9A	109.2
C8B—C3A—C4	120.34 (11)	N2—C9—H9B	109.2
C8B—C3A—C3	117.73 (11)	C10—C9—H9B	109.2
C4—C3A—C3	121.91 (11)	H9A—C9—H9B	107.9
C5—C4—C3A	119.98 (11)	C9—C10—C11	109.44 (9)
C5—C4—H4	120.0	C9—C10—H10A	109.8
C3A—C4—H4	120.0	C11—C10—H10A	109.8
C4—C5—C5A	120.08 (11)	C9—C10—H10B	109.8
C4—C5—H5	120.0	C11—C10—H10B	109.8
C5A—C5—H5	120.0	H10A—C10—H10B	108.2
C8A—C5A—C5	120.67 (11)	N1—C11—C10	112.49 (10)
C8A—C5A—C6	118.17 (11)	N1—C11—H11A	109.1
C5—C5A—C6	121.14 (11)	C10—C11—H11A	109.1
C7—C6—C5A	120.61 (11)	N1—C11—H11B	109.1
C7—C6—H6	119.7	C10—C11—H11B	109.1
C5A—C6—H6	119.7	H11A—C11—H11B	107.8
			
C8B—N1—C1—O1	−178.75 (11)	C8—N2—C8A—C8B	172.78 (10)
C11—N1—C1—O1	14.27 (16)	C9—N2—C8A—C8B	−22.26 (15)
C8B—N1—C1—C2	3.94 (16)	C5—C5A—C8A—N2	−168.25 (11)
C11—N1—C1—C2	−163.05 (10)	C6—C5A—C8A—N2	9.93 (15)
O1—C1—C2—C3	−172.18 (12)	C5—C5A—C8A—C8B	9.86 (16)
N1—C1—C2—C3	5.07 (17)	C6—C5A—C8A—C8B	−171.97 (10)
C1—C2—C3—C3A	−5.67 (19)	C1—N1—C8B—C3A	−12.22 (16)
C2—C3—C3A—C8B	−2.60 (17)	C11—N1—C8B—C3A	154.55 (10)
C2—C3—C3A—C4	175.74 (11)	C1—N1—C8B—C8A	169.50 (10)
C8B—C3A—C4—C5	1.19 (17)	C11—N1—C8B—C8A	−23.73 (15)
C3—C3A—C4—C5	−177.11 (11)	C4—C3A—C8B—N1	−167.08 (10)
C3A—C4—C5—C5A	−8.16 (17)	C3—C3A—C8B—N1	11.29 (16)
C4—C5—C5A—C8A	2.53 (17)	C4—C3A—C8B—C8A	11.25 (16)
C4—C5—C5A—C6	−175.58 (11)	C3—C3A—C8B—C8A	−170.38 (10)
C8A—C5A—C6—C7	−2.72 (17)	N2—C8A—C8B—N1	−20.18 (16)
C5—C5A—C6—C7	175.44 (11)	C5A—C8A—C8B—N1	161.76 (10)
C5A—C6—C7—C8	−5.58 (18)	N2—C8A—C8B—C3A	161.53 (11)
C8A—N2—C8—O2	178.52 (10)	C5A—C8A—C8B—C3A	−16.53 (15)
C9—N2—C8—O2	13.30 (16)	C8—N2—C9—C10	−109.99 (11)
C8A—N2—C8—C7	1.04 (16)	C8A—N2—C9—C10	84.27 (12)
C9—N2—C8—C7	−164.18 (10)	N2—C9—C10—C11	−44.99 (13)
C6—C7—C8—O2	−170.97 (12)	C1—N1—C11—C10	−108.30 (11)
C6—C7—C8—N2	6.44 (17)	C8B—N1—C11—C10	84.22 (12)
C8—N2—C8A—C5A	−9.19 (16)	C9—C10—C11—N1	−42.52 (13)
C9—N2—C8A—C5A	155.77 (10)		
